# A Genomewide Scan for Genetic Structure and Demographic History of Two Closely Related Species, *Rhododendron dauricum* and *R. mucronulatum* (*Rhododendron*, Ericaceae)

**DOI:** 10.3389/fpls.2020.01093

**Published:** 2020-07-17

**Authors:** Baiming Yang, Guoli Zhang, Fengping Guo, Manqi Wang, Huaying Wang, Hongxing Xiao

**Affiliations:** ^1^ Key Laboratory of Molecular Epigenetics of Ministry of Education, Northeast Normal University, Changchun, China; ^2^ Changchun Guoxin Modern Agricultural Technology Development Co., Ltd., Changchun, China; ^3^ Biology Group, No. 30 Middle School of Shenyang, Shenyang, China

**Keywords:** specific-locus amplified fragment, nucleotide differentiation, gene flow, niche simulation, climate changes

## Abstract

Understanding the processes of divergence and speciation is an important task for evolutionary research, and climate oscillations play a pivotal role. We estimated the genetic structure and demographic history of two closely related species of *Rhododendron*, *R. dauricum*, and *R. mucronulatum*, distributed in northeastern China using 664,406 single nucleotide polymorphic loci of specific-locus amplified fragment sequencing (SLAF-seq) and 4 chloroplast DNA (cpDNA) fragments, sampling 376 individuals from 39 populations of these two species across their geographic distributions. The geographical distribution of cpDNA haplotypes revealed that *R. dauricum* and *R. mucronulatum* have different spatial genetic structures and haplotype diversity. Analysis of molecular variance (AMOVA) results showed that these two species have significant genetic differentiation and that the phylogeny demonstrates that these two species clustered a monophyletic group based on SLAF data, respectively, but not in cpDNA data. The evidence of significant gene flow was also detected from *R. mucronulatum* to *R. dauricum*. A deep divergence between the two species was observed and occurred during the early Oligocene. The niche models showed that the two species have different demographic histories. Thus, our results imply that geography and climate changes played important roles in the evolutionary process of *R. dauricum* and *R. mucronulatum*, and although there was an interspecific gene flow, the divergence was maintained by natural selection.

## Introduction

Past climate oscillations and historical tectonism had a huge impact on the genetic structure and demographic histories of many species, even triggering divergence and speciation (Milne, 2006). Understanding the factors that promote species divergence is of major interest in ecological and evolutionary research (Papadopulos et al., 2011; Butlin et al., 2012). Species divergences are frequently driven by geographic isolation, environmental heterogeneity (ecological speciation) or a combination of both (Orr and Smith, 1998; Schluter, 2001; Nosil, 2012). Geographic isolation is generally considered an allopatric speciation, where gene flow among splitting populations is disrupted by physical barriers, and genetic divergence occurs between taxa by local adaptation, mutation, and genetic drift (Coyne and Orr, 2004). In contrast, under ecological speciation, divergence is driven by divergent natural selection between environments, giving rise to reproductive isolation between subsets of a single population by adaptation to different environments or ecological niches. (Schluter, 2000; Schluter, 2001; Rundle and Nosil, 2005).

Gene flow played an important role in the evolution of species, having both positive and negative effects on adaptation (Palme et al., 2004). Itis expected due to secondary contact when physical barriers disappear (under allopatric speciation) or range expansions and contractions in response to climatic fluctuation, which is generally regarded as a force counteracting population divergence (Runemark et al., 2012). When the rate of gene flow between species exceeds that among populations within the species, hybridization might occur. These phenomena have been demonstrated not only in animals (Taylor et al., 2005; Seehausen, 2006; Webb et al., 2011), but also in plants (Marczewski et al., 2015; Ma et al., 2016). Interspecies gene flow after speciation can reduce genetic differentiation among species and even achieve homogenization (Vonlanthen et al., 2012; Bhat et al., 2014). However, when the homogenization of interspecific gene flow is weaker than the disproportionation of natural selection, species genetic differentiation may still be maintained (Rundell and Prince, 2009; Ribera et al., 2011).

*Rhododendron* (Ericaceae L.) is a taxonomically complex genus with approximately 1,000 species (Chamberlain et al., 1996) distributed in Himalayan flora. It is one of the major genera of China and includes approximately 571 species, of which 402 species are endemic to China (Wu et al., 2003; Fang et al., 2005). Northeast Asia is an area with complex topographic and climatic gradients (Lopez-Pujol et al., 2011), and recent research supports a northeastern Asian origin of *Rhododendron* (Shrestha et al., 2018). Among *Rhododendron* species, *Rhododendron* subgen. *Rhodorastrum*, including four taxa, *R. ledebourii*, *R. dauricum*, *R. mucronulatum*, and *R. sichotense*, are distributed in this area. The four species exhibit similar phenotypic characteristics, such as leaf, petiole and flower shapes, thus making taxonomy difficult. *R. ledebourii*, *R. mucronulatum*, and *R. sichotense* were regarded as populations, varieties or subspecies of *R. dauricum* in previous studies (Voroshilov, 1982; Mazurenko and Hohryakov, 1991; Koropachinskii and Vstovskaya, 2002). In contrast, phylogenetic analysis supports the species status of these four species-based cpDNA regions (Tikhonova et al., 2012; Polezhaeva et al., 2018). Additionally, two well-distinguished groups were determined for these four species, including *R. ledebourii* and *R. dauricum* as the Siberian group and *R. sichotense* and *R. mucronulatum* as the Far Eastern group (Polezhaeva et al., 2018), demonstrating good agreement with the geographical positions of the species collected. However, a main haplotype of *R. mucronulatum* was clustered with the haplotypes of *R. sichotense*, and the two species were joined together based on SAMOVA, Furthermore, samples were only from Russia and located in Northeast China (NEC) were lacking. In addition, phylogenetic reconstruction of the genus *Rhododendron* based on the ITS region showed that *R. dauricum* and *R. mucronulatum* were sister species (Kutsev and Karakulov, 2010; Baranova et al., 2014).

**Figure 1:**
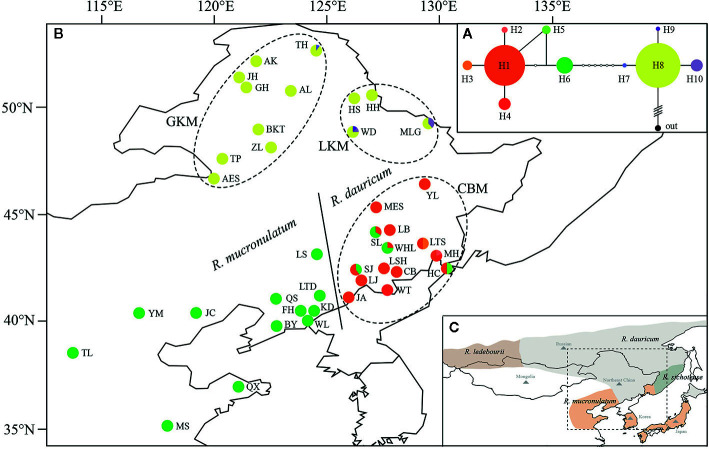
**(A)** The Network of haplotypes in *R. dauricum*. The size of haplotype circle represents relative frequency. **(B)** Geographical distribution of haplotypes within *R. dauricum* and *R. mucronulatum*. The regions of GKM, LKM, and CBM were indicated by dotted ellipses. The populations of *R. dauricum* and *R. mucronulatum* were distinguished by solid line. **(C)** The distribution of four species in subgen. *Rhodorastrum*. It was drawn based on Polezhaeva (2018) and the sites information collected in ecological niche model simulation. Each color represents a species.

These four species have different geographical distribution, covering Mongolia, Northeast China, Japan, Korean Peninsula, and Russian (including the southern part of western Siberia, eastern Siberia, and the Russian Far East), and Northeast China (NEC) is in the center of these areas (**Figure 1C**), among which *R. dauricum* is widely distributed. *R. dauricum* and *R. mucronulatum* have different geographical distribution in NEC, although they both occur along mountain slopes. *R. dauricum* is distributed along the Great Khingan Mountains (GKM), Lesser Khingan Mountains (LKM) and Changbai Mountain (CBM), while *R. mucronulatum* is found in the south of the Changbai Mountain and other locations in North China.

Therefore, in this study, to test the genetic divergence and demographic history of *R. dauricum* and *R. mucronulatum* driven by possible roles of geological or climate events, we first combined specific-locus amplified fragment sequencing (SLAF-seq) and cpDNA markers. We assessed the genetic diversity, phylogeny, and genetic structures of the two species. The divergence time and ecological niche modeling were also tested to infer the possible population demographic history in light of past climate changes. In addition, gene flow between the species was also detected to further elucidate the role of either climate oscillations or geological processes in shaping nucleotide variation and triggering divergence. Together, these analyses not only provide insights into the evolutionary history of two closely related species but also contribute to understanding biodiversity in northern and northeastern China.

## Materials and Methods

### Plant Materials and Sequencing

On the basis of 23 populations in Jiang et al. (2016), we have newly collected some populations and individuals of *R. dauricum*. After removing and merging some populations that were close to each other or for which the haplotypes were completely consistent, we newly added 127 *R. dauricum* individuals in this study. Of these, 88 individuals were from newly added 9 populations and 39 were extensions to the existing populations. Finally, there were a total of 247 *R. dauricum* individuals in 27 populations (**Table 1**). Moreover, we newly collected 12 populations of 129 individuals with *R. mucronulatum* across all the current relevant regions in China. 5 to 23 individuals for each population, only one individual for LB. We distinguished these two species mainly based on geographical distribution and morphological characteristics. Firstly, *R. dauricum* is mainly distributed in Inner Mongolia, Heilongjiang and Jilin Provinces of China, and *R. mucronulatum* is mainly distributed in Liaoning Province, Hebei Province, Beijing, Shandong Province, and northern Jiangsu Province. Secondly, the main differences in morphology between the two are the characteristic of scales on the leaves abaxial surface and whether there are hairs on the branchlets. The leaves abaxial surface of *R. dauricum* densely covered with scales, imbricate or adjacent to each other, spacing 1/2 or 1.5 times their diameter, and branchlets pilose; the spacing of the scales in *R. mucronulatum* was two to four times its diameter and branchlets glabrous. Sampled individuals were located at least 50 m apart. Additionally, samples of *R. micranthum* and *R. schlippenbachii* were also collected to serve as outgroups for cpDNA phylogenetic analyses. All fresh leaf materials were desiccated in silica gel, and voucher specimens were stored in the Northeast Normal University Herbarium (NENU).

**Table 1:** Sampling information and genetic diversity for SLAF data and cpDNA of *R. dauricum* and *R. mucronulatum*.

pop	Location	Long (°E)	Lat (°N)	size	S	h	Hd	π × 10^−3^	voucher
***R. dauricum***				247 (40)	15	10	0.6610	1.8000 (0.2450)	
GKM									
AES^+^	Aersha, IM	120.562	47.410	20 (2)	0	1	0.0000	0.0000	NENU00042001
TP	Taipingling, IM	120.491	47.488	10 (1)	0	1	0.0000	0.0000	NENU00042002
ZL	Zhalant, IM	122.700	48.083	10	0	1	0.0000	0.0000	NENU00042003
BKT*	Boktu, IM	122.105	48.832	10 (3)	0	1	0.0000	0.0000	NENU00042004
GH	Genhe City, IM	121.492	50.775	5 (1)	0	1	0.0000	0.0000	NENU00042005
JH	Jinhe Town, IM	121.294	51.041	5 (1)	0	1	0.0000	0.0000	NENU00042006
AK	Oakley Hill, IM	122.037	51.838	10 (1)	0	1	0.0000	0.0000	NENU00042007
AL	Ali River, IM	123.533	50.550	5	0	1	0.0000	0.0000	NENU00042008
TH*	Tahe County, HLJ	124.710	52.334	10 (3)	1	2	0.2000	0.0001	NENU00042009
LKM									
HS	Big black mountain, HLJ	126.448	50.241	5 (3)	0	1	0.0000	0.0000	NENU00042010
HH	Heihe, HLJ	127.516	50.233	5	0	1	0.0000	0.0000	NENU00042011
MLG^+^	Maolangou, HLJ	129.771	49.098	18 (3)	1	2	0.5033	0.0002	NENU00042012
WD*	Longmen Shizhai, HLJ	126.335	48.695	8 (3)	2	3	0.4643	0.0002	NENU00042013
CBM									
YL	Yilan County, HLJ	129.568	46.325	9 (3)	0	1	0.0000	0.0000	NENU00042014
MES	Maoer Mountain, HLJ	127.449	45.259	5 (1)	0	1	0.0000	0.0000	NENU00042015
SL*	Shulan City, JL	127.452	44.247	12 (3)	2	2	0.4850	0.0004	NENU00042016
LB	Old white mountain, JL	127.919	44.242	1	–	–	–	–	NENU00042017
WHL*	Weihuling, JL	127.866	43.483	10	2	2	0.3560	0.0003	NENU00042018
LTS*	Camel Mountain, JL	129.540	43.645	10 (2)	1	2	0.5560	0.0002	NENU00042019
MH*	Manhe Village, JL	130.055	43.189	10 (2)	2	3	0.6890	0.0003	NENU00042020
HC*	Jiushaping, JL	130.633	42.417	8 (1)	1	2	0.5710	0.0002	NENU00042021
CB^+^	Changbai Mountain, JL	127.787	42.052	11 (3)	0	1	0.0000	0.0000	NENU00042022
LSH	Lushihe, JL	127.883	42.533	6	0	1	0.0000	0.0000	NENU00042023
WT	Wangtian’e Scenic Area, JL	127.943	41.547	6 (1)	1	2	0.3330	0.0001	NENU00042024
SJ	Triangular Bay, JL	126.433	42.350	5	2	2	0.6000	0.0004	NENU00042025
LJ^+^	Pearl Gate Village, JL	126.156	42.278	23 (3)	0	1	0.0000	0.0000	NENU00042026
JA*	Ji’an, JL	126.283	41.250	10	0	1	0.0000	0.0000	NENU00042027
***R. mucronulatum***				129 (25)	0	1	0.0000	0.0000 (0.1850)	
LTD*	Lao Baldingzi, LN	124.908	41.298	21 (2)	0	1	0.0000	0.0000	NENU00042028
LS*	Sitaizi Forest Farm, JL	124.702	43.189	10 (3)	0	1	0.0000	0.0000	NENU00042029
QS*	Qianshan, LN	123.091	41.589	9	0	1	0.0000	0.0000	NENU00042030
FH*	Phoenix Mountain, LN	124.078	40.413	21 (3)	0	1	0.0000	0.0000	NENU00042031
WL*	Wulong Mountain, LN	124.336	40.255	10 (3)	0	1	0.0000	0.0000	NENU00042032
KD*	Kuandian, JL	124.483	40.500	10	0	1	0.0000	0.0000	NENU00042033
BY*	Bingyugou, LN	122.950	39.983	8	0	1	0.0000	0.0000	NENU00042034
JC*	Sandaogou, LN	119.345	40.583	15 (3)	0	1	0.0000	0.0000	NENU00042035
YM*	Yunmeng Mountain, BJ	116.686	40.555	10 (3)	0	1	0.0000	0.0000	NENU00042036
TL*	Tuoliang Scenic Spot,HB	113.812	38.740	5 (3)	0	1	0.0000	0.0000	NENU00042037
QX*	Leshan Park, SD	121.066	37.226	8 (3)	0	1	0.0000	0.0000	NENU00042038
MS*	Mengshan Forest Park, SD	117.969	35.557	2 (2)	0	1	0.0000	0.0000	NENU00042039
Total				376 (65)	16	10	0.5198	0.0014	

S, number of polymorphic sites (SNPs); h, number of haplotypes; Hd, haplotype diversity; π, nucleotide diversity. *represents the newly added populations of R. dauricum and R. mucronulatum in this study, ^+^represents the populations of individual expansion based on previous studies (the number of newly added: AES, 10; MLG, 8; CB, 9; LJ, 12). The size in brackets represents the individuals used for SLAF; the π in brackets represents the nucleotide diversity based on SLAF. GKM, Great Khingan Mountains; LKM, Lesser Khingan Mountains; CBM, Changbai Mountain; IM, Inner Mongolia; HLJ, Heilongjiang Province; JL, Jilin Province; LN, Liaoning Province; BJ, Beijing; HB, Hebei Province; SD, Shandong Province.

Total genomic DNA was extracted using a modified 4 × CTAB procedure (Doyle and Doyle, 1987). The concentration and quality of genomic DNA were tested by agarose gel electrophoresis (1%) and an ND-1000 spectrophotometer (Thermo Fisher Scientific, USA). Qualified DNA samples were stored at −20°C for polymerase chain reaction (PCR) amplification and high-throughput sequencing.

To obtain the orthologous regions of *R. dauricum* individuals from *R. mucronulatum* samples, we selected four pairs of primers (*trnS-trnG*, *trnS-trnfM*, *TabE-ndhJ*, *trnK-matK*) used in a previous study (Jiang et al., 2016), which were most informative for *R. dauricum* populations. The reagent concentrations, polymerase chain reaction (PCR) process, and sequencing followed Jiang et al. (2016).

### Analyses of cpDNA Sequences

DNA sequence alignment was performed using ClustalX 2.0 (Larkin et al., 2007), and alignments were edited manually in BioEdit 7.0.1 (Hall, 1999) before being concatenated. The number of polymorphic sites (S), nucleotide diversity (π), number of haplotypes (h), and haplotype diversity (Hd) were calculated by DnaSP v5 (Librado and Rozas, 2009). We also calculated Tajimas’ D (Tajima, 1989), Fu and Li’ s D and F (Fu and Li, 1993) separately for the two species using this software to test how well the data conformed to the neutral model of evolution. The population differentiation statistic (F_ST_) (Wright, 1984) was calculated by AMOVA in ARLEQUIN 3.5 (Excoffier et al., 2005). Furthermore, the cpDNA haplotype, representing unique DNA sequences or alleles separated by mutational steps, was constructed by TCS v1.2.1 (Clement et al., 2000) with genetic distance and statistical parsimony methods. We chose ‘Gaps = missing’ and set the Fix Connection Limit to 50 steps to ensure that all haplotypes were connected to each other. In order to compare with the phylogenetic tree constructed by SLAF data, we chose the same individuals as SLAF to construct the phylogenetic tree based on cpDNA. A maximum likelihood (ML) tree was constructed in RAxML 8.2.9 software (Stamatakis et al., 2008) under GTRGAMMA selected by jModeltest 2.0 (Darriba et al., 2012) based on Akaike information criterion (AIC) values and bootstrap supports for clades calculated using 1000 bootstrap replicates.

### SLAF Sequencing, Read Alignment and SNP Calling

To obtain all haplotypes of cpDNA and represent the distribution of the two species as evenly as possible, we selected 65 individuals (including 40 *R. dauricum* samples and 25 *R. mucronulatum* samples) for subsequent SLAF sequencing (**Table 1**). Samples of *R. redowskianum* and *R. delavayi* were used as outgroup. The genome of *R. delavayi* (ftp://parrot.genomics.cn/gigadb/pub/10.5524/100001_101000/100331/) was selected as the reference genome for enzyme cutting site prediction. After strict filtering, *Hae*III and *Hpy*166II restriction enzymes were finally used to digest the qualified DNA of the sampled individuals according to the results of prerestriction enzyme digestion. To evaluate the accuracy of the enzyme digestion experiment, *Oryza sativa* subsp. *indica* was used as a control to ensure the effectiveness of the enzyme-cutting scheme. The obtained SLAF tags with an A-tail added to the 3′ ends were ligated with Dual-index sequencing adaptors, amplified by PCR, screened, and then used to construct the SLAF library (for detailed processes of the SLAF library construction, refer to the methods of Sun et al. (2016). The selected tags were then subjected to pair-end sequencing using an Illumina Hiseq™ 2500 at Biomarker Technologies Corporation in Beijing. Sequencing insert size was 314 to 414 bp and paired-end reads were 126 bp in length.

Clear reads were aligned against the reference genome using Burrows-Wheeler Aligner (BWA) (Li and Durbin, 2009) with parameters defined as missed match = 3; opening gap = 11 and gap extension = 4. False alignment was always detected near Indels; therefore, local alignment was performed. The Genome Analysis Toolkit (GATK) (DePristo et al., 2011) and Sequence Alignment/Map tools (SAMtools) (Li et al., 2009) were used for variant calling under the default setting. Raw SNPs were filtered with a minimum depth (DP) of 3 and minimum mapping quality (MQ) of 30 by our custom Perl scripts, and then PLINK 2 (Purcell et al., 2007) was used to further filter with the minor allele frequency (MAF) of 0.04 and maximum missing rate of 0.5, and SNPs that were out of Hardy-Weinberg equilibrium (HWE) were removed with a p-value threshold < 0.01. Later, considering the linkage disequilibrium (LD) between SNPs, we used the -r2 function in PLINK to quantify pairwise LD between all pairs of SNPs located within 1000 kb of each other, and the results showed that the LD of both species decayed rapidly (**Supplementary Figure S1**). Thus, we did not further filter SNPs according to LD. All non-biallelic SNPs, which may be caused by sequencing, were filtered using our custom Perl scripts. The final screened SNPs were subjected to subsequent data analyses.

### Genetic Diversity, Phylogeny, and Population Structure Analyses

The genetic diversity parameter π and the neutral test of Tajima’s D, Fu and Li’ s D and F values were calculated by PopGenome in R (Bastian et al., 2014) based on SNPs. F_ST_ was also calculated by AMOVA in ARLEQUIN 3.5. The ML tree was constructed in the same way as above. The population structure analyses were performed using ADMIXTURE software (Alexander et al., 2009). The number of subgroups (K value) was set as 1 to 10 in advance for clustering, and the clustering results were cross-verified to determine the optimal number of subgroups according to the valley value of the cross-validation error rate. We further performed principal component analysis (PCA) to determine the clustering status of samples using EIGENSOFT (Price et al., 2006).

### Divergence Time and Gene Flow

A Bayesian relaxed molecular clock approach was used to estimate species divergence time by MCMCTREE in PAML (Yang, 2007). When using previously published calibration times, the split of *Oryza sativa* (https://phytozome.jgi.doe.gov/pz//portal.html#!info?alias=Org_Osativa) and *Arabidopsis thaliana* (https://phytozome.jgi.doe.gov/pz/portal.html#!info?alias=Org_Athaliana_er) was fixed as 130~200 Mya, and the split of *R. delavayi* and *Actinidia chinensis* (http://bioinfo.bti.cornell.edu/cgi-bin/kiwi/download.cgi) was estimated to be in the range of 56.1~120.8 Mya (Zhang et al., 2017). The 150 bp sequences before and after the SNPs were used for BLASTN (Altschul et al., 1990) against the reference genomes with an E-value threshold of 1e-3, and then obtained the sequence with a total length of 12,936,348 bp as an input file. *R. dauricum* and *R. mucronulatum* individuals were selected from populations TP and MS, respectively, based on the cpDNA haplotype. The data set was modeled under a correlated rates clock, and the HKY85 nucleotide substitution model was determined by ModelFinder (Kalyaanamoorthy et al., 2017). The first 2000 iterations were discarded as burn-in, and every 10 iterations were sampled until 20000 samples were obtained. Furthermore, to account for gene flow among the groups, we used TREEMIX 1.12 (Pickrell and Pritchard, 2012), which infers patterns of population splitting and mixing accessing the covariance structure of allele frequencies between populations and performing a Gaussian approximation for genetic drift.

### Ecological Niche Modeling

MaxEnt software (Phillips et al., 2006) was used to predict the distribution models for *R. dauricum* and *R. mucronulatum* in the last interglacial (LIG) (0.14–0.12 Mya), the last maximum glacial (LGM) (0.02–0.018 Mya) and current time based on modern distribution records and bioclimatic variables. The occurrence data of species were mainly collected from the following three sources: Chinese Virtual Herbarium (CVH) (http://www.cvh.ac.cn/) and relevant literature; Global Biodiversity Information Facility (GBIF) (http://www.gbif.org); and sampling point information obtained from our field survey. 316 sites of *R. dauricum* and 214 sites of *R. mucronulatum* worldwide were obtained. Then, the distribution data obtained in these three ways were manually checked and proofread, and duplicate records within each 2.5-arc-minute cell and artificially planted species distribution points were removed. Finally, 238sites of *R. dauricum* and 214 sites of *R. mucronulatum* worldwide were obtained.

The 19 bioclimatic environmental variables were downloaded from the world climate data website (http://www.worldclim.org) with a 30 arc-second resolution for the present and LIG and 2.5 arc-minute for LGM. Then, ArcGIS software (Johnston et al., 2001) was used to perform pairwise correlations among the 19 variables. If r > 0.8, only one of the variables was selected based on the relative contribution to the model to minimize biased fitting of niche models. Accordingly, seven environmental variables were used to simulate species distribution areas for *R. dauricum* and *R. mucronulatum*. Model evaluation statistics were produced from 15 replicate runs with 60% of the data used for training and 40% for model testing. Finally, the accuracy of the predicted distribution results was tested by the ROC curve analysis method, and the AUC value (the area under the ROC curve) was obtained. The closer the AUC value is to 1, the farther it is from a random distribution, the greater the correlation between environmental variables and the predicted geographical distribution of species, and the more accurate the model prediction results will be.

## Results

### Genetic Diversity and Neutrality Test

The concatenated cpDNA sequences (including indels) had an aligned length of 2,711 bp, in which 15 SNPs and 10 haplotypes were identified (**Table 1**). Excluding the 5 haplotypes published by Jiang et al. (2016), the new 5 haplotypes sequences were deposited in the GenBank database (MT603719–MT603738). In 376 individuals from 39 populations, the haplotype diversity (Hd) and nucleotide diversity (π) ranged from 0 to 0.5198 and from 0 to 0.0014, respectively. Independently, 15 SNPs and 10 haplotypes were identified in *R. dauricum* populations, and the Hd was 0.6610 and π was 0.0018. The results of the neutrality test showed no significant positive or negative value (Tajima’s D = 1.3348, Fu and Li’s D = −0.1766, Fu and Li’s F = 0.3798; P > 0.1). In contrast, only one haplotype (H6) was detected in *R. mucronulatum* populations and shared with *R. dauricum*; that is, there was no variation. The pairwise F_ST_ values (0.3541) showed significant genetic differentiation between *R. dauricum* and *R. mucronulatum*.

After SLAF library construction and high-throughput sequencing, a total of 735,337 SLAF tags was developed, and the average depth was 16.33x, which generated 328.74 Mb reads, with a mean Q30 of 92.02% and a GC content of approximately 41.53%. The numbers of SLAF markers in each individual ranged from 95,609 to 282,183. Among the SLAFs that were detected in total, 79,696 SLAFs were mapped to the *R. delavayi* genome and were distributed equally on each chromosome (**Supplementary Figure S2A**). Then, a total of 6,514,510 SNPs across the 65 accessions were identified by both GATK and SAMtools, which were considered to be reliable and equally well spread across all chromosomes (**Supplementary Figure S2B**). After various filtering, 664,406 SNPs were finally selected for downstream analyses. The SLAF data have been submitted to the Sequence Read Archive (SRA) database in the NCBI and the accession number was PRJNA589346. The average nucleotide diversity of *R. dauricum* was 0.2450 × 10^−3^, which was lower than that of the cpDNA (**Table 2**). In contrast, the average nucleotide diversity of *R. mucronulatum* (0.1850 × 10^−3^) was higher than that of cpDNA. The results of the neutrality test showed a significant positive value (Tajima’s D = 0.8196, Fu and Li’ s D = 1.5824, Fu and Li’ s F = 1.5494 in *R. dauricum*; Tajima’s D = 0.6661, Fu and Li’ s D = 0.9743, Fu and Li’ s F = 0.9989 in *R. mucronulatum*; P < 0.1), which might indicate that these species experienced a bottleneck in their evolutionary history or balancing selection of these loci.

**Table 2:** Summary results of AMOVA and F_ST_.

Locus	cpDNA		SLAF	
	% of variation	P	% of variation	P
Among species	28.57	<0.01	57.98	<0.01
Among populations within species	68.06	<0.01	18.59	<0.01
Within populations	3.37	<0.01	23.43	<0.01
F_ST_ Among species	0.3541		0.2886	

### Population Structure

The topology obtained from TCS based on cpDNA was used to infer relationships among the 10 cpDNA haplotypes (**Figure 1A**), of which H1, H6, H8 were the most frequent haplotypes and were located in the center of the haplotype network. However, these three major haplotypes are geographically segregated: H1 from the Changbai Mountains populations of *R. dauricum*, H6 from *R. mucronulatum* populations and H8 exclusive to the Great/Lesser Khingan Mountains populations of *R. dauricum*. Eight mutational steps differentiate H6 from H8, while only two steps separate haplotype H1 from H6 (**Figure 1A**). In addition, some low-frequency haplotypes were found in a few populations of *R. dauricum*.

**Figure 2:**
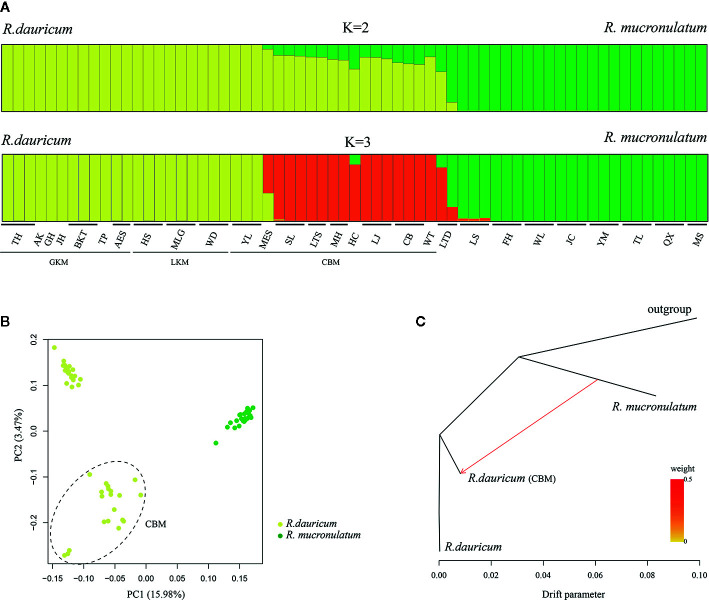
**(A)** Bayesian clustering of all *R. dauricum* and *R. mucronulatum* individuals. Each rectangle represents an individual. The population and region codes were placed at the bottom. **(B)** Plot of principle component analysis (PCA). The dotted ellipse represents the individuals of *R. dauricum* from the Changbai Mountains. **(C)** TREEMIX analysis of population splitting and migration events. Colored arrows represent migration events from *R. mucronulatum* to *R. dauricum* (CBM) with color ramp indicating the magnitude of gene flow.

To further understand the evolutionary history of the two species, we used a Bayesian clustering algorithm to estimate the population genetic structure. The results of ADMIXTURE showed that K=2 was the optimal value when the cross-validation (CV) errors were applied(**Supplementary Figure S3**). When K=2, *R. dauricum* and *R. mucronulatum* clustered into distinct groups and showed majority populations collected from the Changbai Mountains (MES, SL, LTS, MH, HC, LJ, CB, WT) of *R. dauricum* with some admixture from *R. mucronulatum* (**Figure 2A**). When K=3, the admixture *R. dauricum* samples were separate. PCA results also showed that samples from *R. dauricum* and *R. mucronulatum* clustered into different groups by PC1, while the samples of *R. dauricum* from Great/Lesser Khingan Mountains and Changbai Mountains were clustered into distinct groups by PC2 (**Figure 2B**), consistent with the result of the ADMIXTURE analysis.

### Phylogeny and Divergence Time

**Figure 3:**
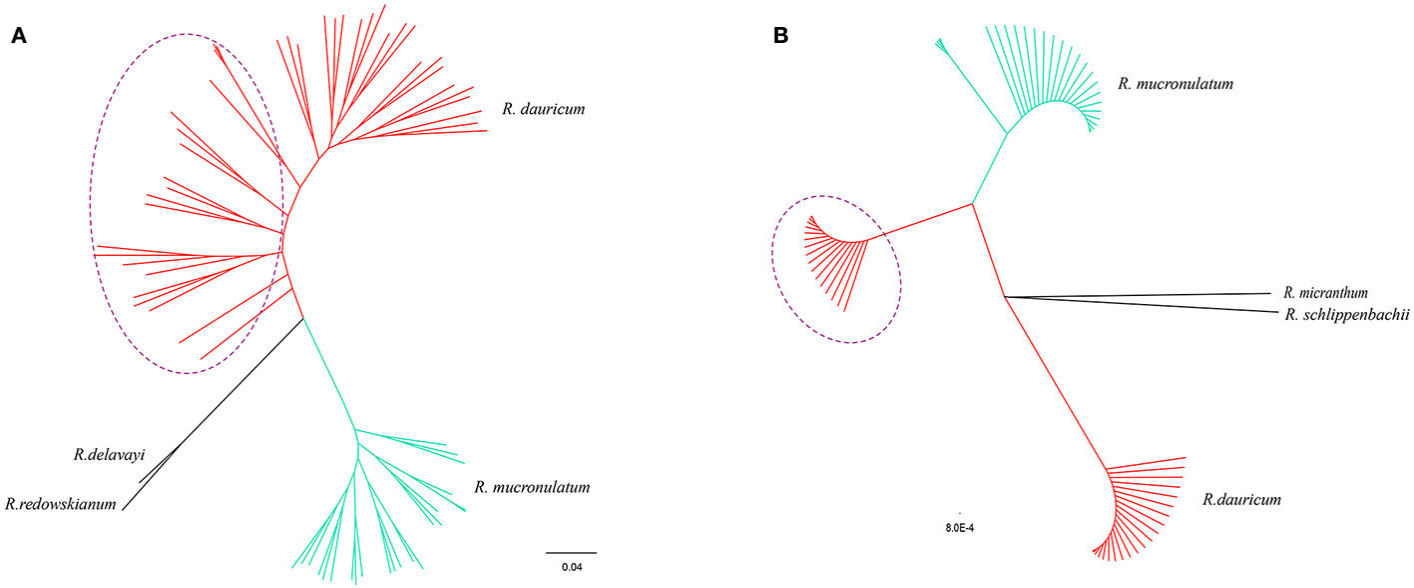
**(A)** Phylogenetic trees using the maximum-likelihood (ML) method on the basis of 664,406 SNPs. **(B)** The maximum-likelihood (ML) tree based on 4 cpDNA (the same individuals as SLAF). Bootstrap support values all >90%. The red represents the individuals of *R. dauricum*; the green represents the individuals of *R. mucronulatum*. The purple dotted ellipse represents the individuals from the Changbai Mountains.

The maximum-likelihood (ML) phylogenetic tree of all samples based on the 664,406 SNPs showed that *R. dauricum* and *R. mucronulatum* formed a well-supported clade, respectively. (**Figure 3A**). In the *R. dauricum* clade, the individuals from Changbai Mountain formed a subclade, and others formed another clade, with a high support value (bootstrap values > 90%). However, the ML tree based on cpDNA (**Figure 3B**, note that it represents a bootstrap consensus tree and therefore does not show all individual cpDNA haplotypes) presented multiple incongruences with the phylogenetic tree based on SLAF data. The cpDNA phylogeny identified three main lineages: *R. mucronulatum*, *R. dauricum* from Changbai Mountain and *R. dauricum* from elsewhere. *R. dauricum* did not constitute a monophyletic group.

**Figure 4:**
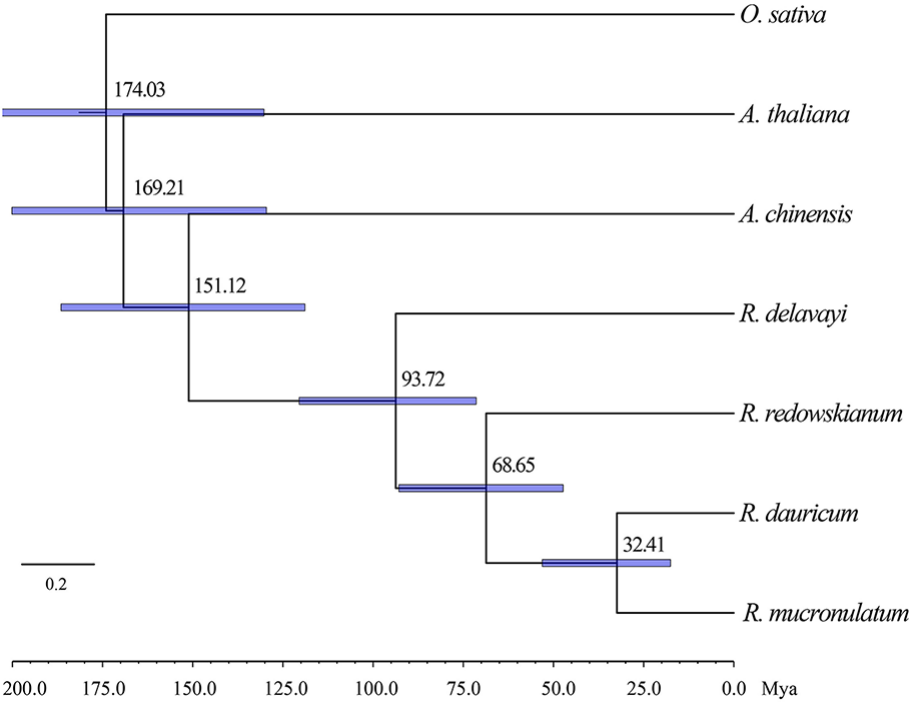
Phylogenetic tree showing result from divergence time analysis using MCMCTREE. Numbers at nodes are divergence age, timescale is in million years ago (Mya). A geological time scale is shown below the tree. Node bars indicate the intervals of 95% HPD.

Based on the two previously published calibration time points, the divergence time was calculated based on the maximum-likelihood (ML) tree using MCMCTREE (**Figure 4**), which pointed toward the divergence time of outgroup *R. redowskianum* from the most recent common ancestor of *R. dauricum*, and *R. mucronulatum* was dated to have occurred 68.65 Mya (95% HPD interval: 71.42–120.48 Mya), while the divergence time of *R. dauricum* and *R. mucronulatum* was estimated at 32.41 Mya (95% HPD interval: 17.54–53.11 Mya) during the early Oligocene. This was in effect also the divergence time between the Siberian and the Far Eastern clades of subgen. *Rhodorastrum*.

### Genetic Differentiation and Gene Flow

Our hierarchical AMOVA showed that the percentage of variation among species was 28.57% and was significant in terms of cpDNA (P < 0.01) (**Table 2**). In general, the variations among species in SLAF were 57.98% higher than cpDNA and were the main source of variation, while the main source of variation in cpDNA was found among populations within species (68.06%). The pairwise F_ST_ values between *R. dauricum* and *R. mucronulatum* showed significant genetic differentiation among species (F_ST_ = 0.3541 in cpDNA; F_ST_ = 0.2886 in SLAF). The results of TREEMIX suggested a higher proportion of gene flow in populations of *R. dauricum* and *R. mucronulatum* from Changbai Mountain (**Figure 2C**), which may be a major reason for the lack of complete species divergence and the shared haplotype (H6) between *R. dauricum* (SJ, SL, and WHL) and *R. mucronulatum* (**Figure 1B**).

### Distribution Model Analysis

**Figure 5:**
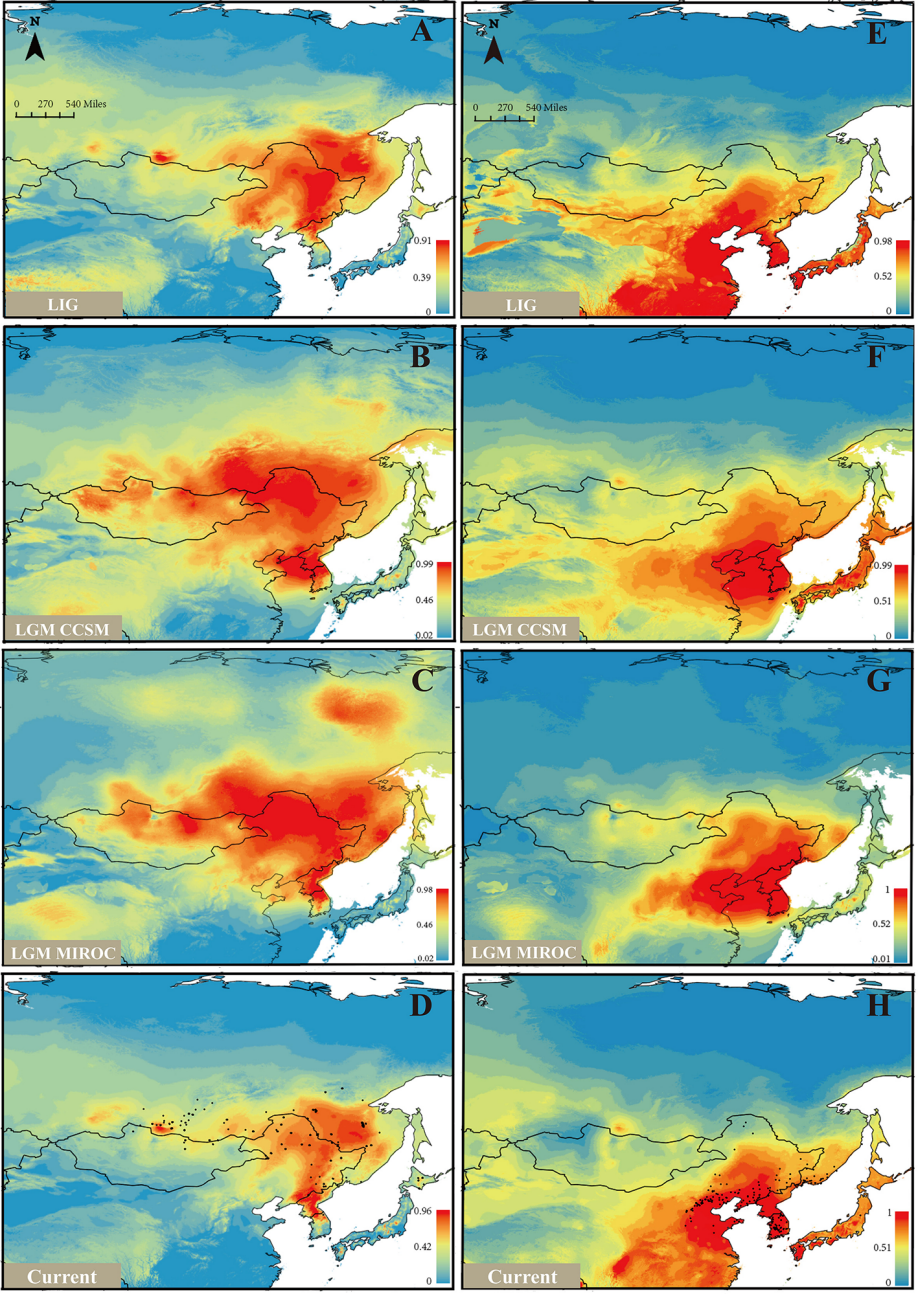
Ecological niche models (ENMs) for *R. dauricum* and *R. mucronulatum*. **(A–D)** represents the distribution of *R. dauricum*; **(E–H)** represents the distribution of *R. mucronulatum*. The black dots in D and H represent the sites of *R. dauricum* and *R. mucronulatum* used for simulation.

Based on the model fitting of the *R. mucronulatum* and *R. dauricum* distribution areas by MaxEnt software, the high ROC values (*R. dauricum*: 0.896, 0.881, 0.881, 0.909; *R. mucronulatum*: 0.910, 0.945, 0.959, 0.948) indicate the good accuracy of our model predictions. *R. mucronulatum* in China is mainly distributed in Liaoning, Beijing, Hebei, and Shandong, and the simulated result of the current potential distribution areas is basically consistent with the current distribution areas (**Figure 5H**), although some areas of southern China were also predicted to be suitable. Based on the paleoclimate information simulation, this species had a relatively large distribution range in the LIG period (**Figure 5E**), while in the subsequent LGM period (**Figures 5F, G**), both the CCSM and MIROC models showed that the distribution areas shrank and migrated to the southeast. From LGM until now (**Figures 5F–H**), the distribution range had neither obvious shrinkage or expansion. In contrast, *R. dauricum* populations showed the opposite trend, with a relatively small distribution range in the LIG (**Figure 5A**) and expansion during the LGM (**Figures 5B, C**), with subsequent shrinkage when the climate was warm (**Figure 5D**). This is probably related to the fact that *R. dauricum* is a cold-tolerant plant.

## Discussion

### Differences in Genetic Diversity Between *R. dauricum* and *R. mucronulatum*

Based on the genetic diversity values obtained in the present study, the total nucleotide diversity (π = 0.0014, **Table 1**) is relatively high at the subgen. *Rhodorastrum* level, and the haplotype diversity (Hd = 0.5198) is at a medium level. These two species showed different levels of nucleotide diversity based on SLAF and cpDNA data. The analysis results of SLAF data showed that *R. dauricum* and *R. mucronulatum* have similar nucleotide diversity, but a higher level of species-wide nucleotide diversity was found in *R. dauricum*, consistent with a previous report (Jiang et al., 2016) and that of *R. mucronulatum* was zero in cpDNA. The nucleotide diversity of *R. dauricum* was higher than that of other species of *Rhododendron*, such as *R. simsii* (π = 1.06 × 10^−3^) (Li et al., 2012). In addition, *R. dauricum* contained all 10 haplotypes, while *R. mucronulatum* had only one haplotype (H6) and shared it with *R. dauricum*. Compared to other temperate species, such as *R. delavayi* (Hd = 0.5570) (Sharma et al., 2014), *R. simsii* (Hd = 0.749) (Li et al., 2012), and *Platycrater arguta* (Hd = 0.8820) (Qiu et al., 2009), *R. dauricum* was in the middle (Hd = 0.6610), but fewer than 170 angiosperms indicate cpDNA haplotype diversity (Hd = 0.6700) (Petit et al., 2005).

As noted by Wright and Gaut (2005), a series of factors may contribute to genetic diversity in plant species. The niche simulation analysis showed that *R. dauricum* and *R. mucronulatum* occupy different niches (**Figures 5D, H**), so climate may be an important driver that causes genetic diversity between the two species (Wang et al., 2015). For the extreme situation where *R. mucronulatum* has only one cpDNA haplotype, similarly low cpDNA diversity has been found in *Acer mono* (Guo et al., 2014) and *Juglans mandshurica* (Bai et al., 2016) distributed in NEC. These provide evidence that many of these northern populations may have experienced a bottleneck during LGM. Also, the limitation of marker sampling cannot be excluded. Furthermore, due to the ornamental value of this species, the influence of human factors on diversity cannot be ignored.

### Oligocene Divergent Event and Demographic Histories of Two Species

Climate changes and tectonic events have had a profound influence on the evolution of different lineages and have often been associated with rapid divergence or speciation (Milne and Abbott, 2002; Milne, 2006; Mao et al., 2012; Zou et al., 2013). Previous studies showed drastic global cooling after the early Eocene Climatic Optimum (EECO; 52–50 Ma) (Zachos et al., 2001). Our study inferred that the divergence of *R. dauricum* and *R. mucronulatum* occurred at 32.41 Mya (i.e., the divergence between the Siberian and the Far Eastern clades, **Figure 4**). This may be driven by the global dramatic climate cooling and reduced sea level (Ivany et al., 2000; Katz et al., 2008; Liu et al., 2009; Buerki et al., 2013) during the late Eocene to the early Oligocene (40–30 Mya). In addition, the separation of most continents and the collision between the plates have a direct or indirect impact on speciation (Hall, 2009). During the North China tectonic period of the Middle Eocene-Oligocene (52–23.3 Mya), the Pacific plate displaced in the NWW (West-Northwest) direction. At approximately 34 Mya, the direction of motion suddenly changed from NNW (West-Northwest) to NWW, causing severe deformation in the eastern part of the Eurasian plate, and mainland China was squeezed in an east-west direction, simultaneously resulting in a series of near-south-north folds (Wan and Cao, 1992). Thus, the divergence between Siberian and the Far Eastern clades was likely to be directly impacted by geomorphological changes.

Based on the cpDNA haplotype geographical distribution (**Figure 1**), we found that the two populations of MLG and WD of *R. dauricum* located in LKM have the ancient haplotype H8, special haplotypes H9, H10, and a higher haplotype diversity. The populations of *R. dauricum* in CBM also have special haplotypes and higher haplotype diversity. In addition, the results of the niche model showed that the populations of *R. dauricum* clustered to the LKM and CBM in the LIG, with subsequent local expansion during the LGM. Therefore, it is speculated that multiple refugias were maintained in *R. dauricum.* Li et al. (2012) and Bao et al. (2015) also found similar situations for *R. simsii* and *Pinus koraiensis*. In contrast, *R. mucronulatum* is currently mainly distributed in North China. The niche model showed that its distribution shrank back to the Korean Peninsula during the LGM; thus, the Korean Peninsula may be a glacial refugia for *R. mucronulatum* (Watanabe et al., 2016).

### The Relative Effectiveness of Natural Selection and Gene Flow in the Evolution of the Two Species

Natural selection and gene flow are two important influential factors in the evolution of species. Gene flow generally occurs within a species, but there are numerous examples of interspecific gene flow. Gene flow can achieve homogenizing effects between gene pools (Coyne and Orr, 2004), while natural selection can weaken this effect. When natural selection overcomes the homogenizing effects of gene flow, populations or species are able to evolve separately. Our results of the species tree estimated by TREEMIX suggested a higher proportion of gene flow in populations of *R. dauricum* from *R. mucronulatum* (**Figure 2C**), and the main hybrid zone was observed in the CBM region. Higher levels of migration were suggested from populations of *R. mucronulatum* to populations of *R. dauricum* located in the CBM. The divergence time analysis shows a pre-LGM divergence of the two species (**Figure 4**), and ecological niche modeling revealed the distribution from LGM to now of the two species overlapped in the CBM region (**Figure 5**). Therefore, gene flow may occur due to the overlap of regions (Li et al., 2010; Abbott et al., 2013; Sun et al., 2016).

In the haplotype geographical distribution, the range-edge populations (SJ, SL, and WHL) of *R. dauricum* contained the haplotype (H6) from *R. mucronulatum* (**Figure 1B**). Consistent with this, the phylogeny tree based on cpDNA showed that the *R. dauricum* individuals from CBM did not cluster with others that was assigned to the *R. mucronulatum* clade (**Figure 3B**). This suggests that there may be seed-mediated unidirectional gene flow from *R. mucronulatum* to the populations of *R. dauricum* in the CBM. However, the phylogeny tree based on markers sampled across the SLAF data present the correct topology that *R. dauricum* and *R. mucronulatum* form a monophyly, respectively. In addition, the results of clustering analysis based on the SLAF data (K=2) showed that individuals from the CBM experienced introgression from *R. mucronulatum*, and that group was separate when K=3 (**Figure 2**), but the optimal K value was 2. The AMOVA results showed that the main source of variation occurred among species in the SLAF data. In general, the main source of variation in cpDNA was among populations within species (**Table 2**), which may be due to the gene flow from *R. mucronulatum* increasing genetic differences between populations of *R. dauricum*. Furthermore, the F_ST_ value showed that the genetic differentiation between *R. dauricum* and *R. mucronulatum* was generally significant (**Table 2**). Therefore, all our results suggest that interspecies gene flow exists, but the role of gene flow is not sufficient to offset the disproportionation of natural selection, and the status of the two species has been maintained (Sun et al., 2016). Therefore, we support Tikhonova’s point mentioned above regarding the relationship between the two species that acted as two independent species.

On the other hand, the inconsistency between SLAF and cpDNA also indirectly illustrates the limitations of cpDNA for studying the genetic differentiation and speciation of closely related species, especially the presence of gene flow. Previous research has also demonstrated low species discrimination (24%) for *Rhododendron* based on cpDNA for both coding and noncoding gene regions when used singly or in combination (Li et al., 2011).

## Conclusion

Numerous studies have shown that speciation and diversification cannot be adequately explained only by geographic isolation. Factors such as local selection and gene flow may likely and prevalently influence the divergence and diversification of closely related species. Our study investigated the genetic structure and demographic history of two closely related species distributed in northeastern China and revealed that divergence was strongly influenced by early the Oligocene climatic shift. Despite the deep differentiation of the two species, gene flow was not completely interrupted. The significantly different biogeographic structures indicated that the two species experienced divergent selection since their differentiation and overcame the homogenizing effect of gene flow. Our study provides an additional case of the evolutionary history of two closely related species and contributes to understanding the factors affecting this process.

## Data Availability Statement

The original contributions presented in the study are publicly available. The SLAF data can be found in NCBI : [PRJNA589346], the cpDNA data can be found here: [MT603719-MT603738]. Publicly available datasets were analyzed in this study. This data can be found here: [https://www.ncbi.nlm.nih.gov/genbank/ KT696944-KT697615]. 

## Author Contributions

BY and HX designed the research and wrote the manuscript. GZ conducted data analysis and figures made. FG performed the experiments. GZ, FG, MW, and HW collected experimental samples.

## Funding

This work was supported by the Natural Science Foundation of the Science and Technology Department of Jilin Province (Subject layout project: 20190201184JC).

## Conflict of Interest

Author Baiming Yang was employed by company Changchun Guoxin Modern Agricultural Technology Development Co., Ltd.,.

The remaining authors declare that the research was conducted in the absence of any commercial or financial relationships that could be construed as a potential conflict of interest.

## References

[B1] Abbott R. Albach D. Ansell S. Arntzen J. W. Baird S. J. E. Bierne N. (2013). Hybridization and speciation. J. Evol. Biol. 26, 229–246. 10.1111/j.1420-9101.2012.02599.x 23323997

[B2] Alexander D. H. Novembre J. Lange K. (2009). Fast model-based estimation of ancestry in unrelated individuals. Genome Res. 19, 1655–1664. 10.1101/gr.094052.109 19648217 PMC2752134

[B3] Altschul S. F. Gish W. Miller W. Myers E. W. Lipman D. J. (1990). Basic local alignment search tool. J. Mol. Biol. 215, 403–410. 10.1016/S0022-2836(05)80360-2 2231712

[B4] Bai W. N. Wang W. T. Zhang D. Y. (2016). Phylogeographic breaks within Asian butternuts indicate the existence of a phytogeographic divide in East Asia. New Phytol. 209, 1757–1772. 10.1111/nph.13711 26499508

[B5] Bao L. Ayijiamali K. Bai W. N. Chen R. Z. Wang T. M. Wang H. F. (2015). Contributions of multiple refugia during the last glacial period to current mainland populations of Korean pine (*Pinus koraiensis*). Sci. Rep. 5:18608. 10.1038/srep18608 26691230 PMC4686996

[B6] Baranova T. V. Kalendar R. N. Kalaev V. N. (2014). K voprosu flogenii roda *Rhododendron* L. na osnove posledovatelnosti speisera ITS1-ITS2. Sibirsk Lesn. Zhur. 6, 29–45.

[B7] Bastian P. R. Ulrich W. Sebastian E. R.-O. Martin J. (2014). PopGenome: An Efficient Swiss Army Knife for Population Genomic Analyses in *R* . Mol. Biol. Evol. 31, 1929–1936. 10.1093/molbev/msu136 24739305 PMC4069620

[B8] Bhat S. Amundsen P. A. Knudsen R. Gjelland K. Ø. Fevolden S. E. Bernatchez L. (2014). Speciation reversal in European whitefish (*Coregonus lavaretus* (L.)) caused by competitor invasion. PLoS One 9, e91208. 10.1371/journal.pone.0091208 24626131 PMC3953381

[B9] Buerki S. Forest F. Stadler T. Alvarez N. (2013). The abrupt climate change at the Eocene-Oligocene boundary and the emergence of South-East Asia triggered the spread of sapindaceous lineages. Ann. Bot. 112, 151–160. 10.1093/aob/mct106 23723259 PMC3690995

[B10] Butlin R. Debelle A. Kerth C. Snook R. R. Beukeboom L. W. Cajas R. F. C. (2012). What do we need to know about speciation? Trends Ecol. Evol. 27, 27–39. 10.1016/j.tree.2011.09.002 21978464

[B11] Chamberlain D. F. Hyam R. Argent G. Fairweather G. Walter K. S. (1996). The genus Rhododendron—its classification and synonymy (Edinburgh: Royal Botanical Garden of Edinburgh Press).

[B12] Clement M. Posada D. Crandall K. A. (2000). TCS: a computer program to estimate gene genealogies. Mol. Ecol. 9, 1657–1659. 10.1046/j.1365-294x.2000.01020.x 11050560

[B13] Coyne J. A. Orr H. A. (2004). Speciation (Sunderland, MA: Sinauer Associates).

[B14] Darriba D. Taboada G. L. Doallo R. Posada D. (2012). jModelTest 2: more models, new heuristics and parallel computing. Nat. Methods 9, 772. 10.1038/nmeth.2109 PMC4594756 22847109

[B15] DePristo M. A. Banks E. Poplin R. Garimella K. V. Maguire J. R. Hartl C. (2011). A framework for variation discovery and genotyping using next-generation DNA sequencing data. Nat. Genet. 43, 491–498. 10.1038/ng.806 21478889 PMC3083463

[B16] Doyle J. Doyle J. L. (1987). Genomic plant DNA preparation from fresh tissue-CTAB method. Phytochem. Bull. 19, 11–15.

[B17] Excoffier L. Laval G. Schneider S. (2005). Arlequin (version 3.0): an integrated software package for population genetics data analysis. Evol. Bioinform. 1, 47–50. 10.1177/117693430500100003 PMC2658868 19325852

[B18] Fang M. Y. Fang R. C. He M. Y. Hu L. Z. Yang H. B. Chamberlain D. (2005). Rhododendron (Beijing & St. Louis: Science Press & Missouri Botanical Garden Press).

[B19] Fu Y. X. Li W. H. (1993). Statistical tests of neutrality of mutations. Genetics 133, 693–709. 10.0000/PMID8454210 8454210 PMC1205353

[B20] Guo X. D. Wang H. F. Bao L. Wang T. M. Bai W. N. Ye J. W. (2014). Evolutionary history of a widespread tree species *Acer mono* in East Asia. Ecol. Evol. 4, 4332–4345. 10.1002/ece3.1278 25540694 PMC4267871

[B21] Hall T. A. (1999). BioEdit: a user-friendly biological sequence alignment editor and analysis program for Windows 95/98/NT. Nucleic Acids Symp Ser. 41, 95–98. 10.1021/bk-1999-0734.ch008

[B22] Hall R. (2009). Southeast Asia’s changing palaeogeography. Blumea 54, 148–161. 10.3767/000651909X475941

[B23] Ivany L. C. Patterson W. P. Lohmann K. C. (2000). Cooler winters as a possible cause of mass extinctions at the Eocene/Oligocene boundary. Nature 407, 887–890. 10.1038/35038044 11057663

[B24] Jiang N. Man L. Zhang W. Dong H. X. Wang H. Y. Li M. R. (2016). Chloroplast View of the Population Genetics and Phylogeography of a Widely Distributed Shrub Species, *Rhododendron dauricum* (Ericaceae). Syst. Bot. 41, 626–633. 10.1600/036364416X692343

[B25] Johnston K. Ver H. J. M. Krivoruchko K. Lucas N. (2001). Using ArcGIS geostatistical analyst. Redlands: Esri. 300.

[B26] Kalyaanamoorthy S. M. Wong B. Q. Thomas K. F. Haeseler A. V. Jermiin L. S. (2017). modelfinder: fast model selection for accurate phylogenetic estimates. Nat. Methods. 14, 587–589. 10.1038/nmeth.4285 28481363 PMC5453245

[B27] Katz M. E. Miller K. G. Wright J. D. Wade B. S. Browning J. V. Cramer B. S. (2008). Stepwise transition from the Eocene greenhouse to the Oligocene icehouse. Nat. Geosci. 1, 329–334. 10.1038/ngeo179

[B28] Koropachinskii I. Y. Vstovskaya T. N. (2002). Drevesnye rasteniya aziatskoi Rossii (Novosibirsk: Publisher House of SB RAS).

[B29] Kutsev M. G. Karakulov A. V. (2010). Rekonstruktsiya flogenii roda *Rhododendron* L. (Ericaceae) fory Rossii na osnove posledovatelnostei speiserov ITS1-ITS2. Turczaninowia 13, 59–62.

[B30] Larkin M. A. Blackshields G. N. Brown P. Chenna R. McGettigan P. A. McWilliam H. (2007). Clustal W and clustal X version 2.0. Bioinformatics 23, 2947–2948. 10.1093/bioinformatics/btm404 17846036

[B31] Li H. Durbin R. (2009). Fast and accurate short read alignment with Burrows-Wheeler transform. Bioinformatics 25, 1754–1760. 10.1093/bioinformatics/btp324 19451168 PMC2705234

[B32] Li H. Handsaker B. Wysoker A. Fennell T. Ruan J. (2009). The sequence alignment/map format and SAMtools. Bioinformatics 25, 2078–2079. 10.1093/bioinformatics/btp352 19505943 PMC2723002

[B33] Li J. W. Yeung C. K. L. Tsai P. W. Lin R. C. Yeh C. F. Yao C. T. (2010). Rejecting strictly allopatric speciation on a continental island: prolonged postdivergence gene flow between Taiwan (*Leucodioptron taewanus*, Passeriformes Timaliidae) and Chinese (*L. canorum canorum*) hwameis. Mol. Ecol. 19, 494–507. 10.1111/j.1365-294X.2009.04494.x 20070521

[B34] Li D. Z. Gao L. M. Li H. T. Wang H. Ge S. J. Liu J. Q. (2011). Comparative analysis of a large dataset indicates that internal transcribed spacer (ITS) should be incorporated into the core barcode for seed plants. Proc. Nat. Acad. Sci. U. S. A. 108, 19641–19646. 10.1073/pnas.1104551108 PMC3241788 22100737

[B35] Li Y. Yan H. F. Ge X. J. (2012). Phylogeographic analysis and environmental niche modeling of widespread shrub *Rhododendron simsii* in China reveals multiple glacial refugia during the last glacial maximum. J. Sys. Evol. 50, 362–373. 10.1111/j.1759-6831.2012.00209.x

[B36] Librado P. Rozas J. (2009). DnaSP v5: a software for comprehensive analysis of DNA polymorphism data. Bioinformatics 25, 1451–1452. 10.1093/bioinformatics/btp187 19346325

[B37] Liu Z. H. Pagani M. Zinniker D. DeConto R. Huber M. Brinkhuis H. (2009). Global cooling during the Eocene-Oligocene climate transition. Science 323, 1187–1190. 10.1126/science.1166368 19251622

[B38] Lopez-Pujol J. Zhang F. M. Sun H. Q. Ying T. S. Ge S. (2011). Centres of plant endemism in China: places for survival or for speciation? J. Biogeogr. 38, 1267–1280. 10.1111/j.1365-2699.2011.02504.x

[B39] Ma Y. P. Xie W. J. Sun W. B. Marczewski T. (2016). Strong reproductive isolation despite occasional hybridization between a widely distributed and a narrow endemic *Rhododendron* species. Sci. Rep. 6, 19146. 10. 10.1038/srep19146 26751844 PMC4707479

[B40] Mao K. Milne R. II Zhang L. Peng Y. L. Liu J. Q. Philip T. (2012). Distribution of living Cupressaceae reflects the breakup of Pangea. Proc. Nat. Acad. Sci. U. S. A. 109, 7793–7798. 10.1073/pnas.1114319109 PMC3356613 22550176

[B41] Marczewski T. Chamberlain D. F. Milne R. II (2015). Hybridization in closely related *Rhododendron* species: half of all species-differentiating markers experience serious transmission ratio distortion. Ecol. Evol. 5, 3003–3022. 10.1002/ece3.1570 26357534 PMC4559045

[B42] Mazurenko M. T. Hohryakov A. P. (1991). Ericaceae Juss (St. Petersburg: Sosudistye rasteniya sovetskogo Dalnego Vostoka. Nauka Press).

[B43] Milne R. II Abbott R. J. (2002). The origin and evolution of Tertiary relict floras. Adv. Bot. Res. 38, 281–314. 10.1016/S0065-2296(02)38033-9

[B44] Milne R. II (2006). Northern Hemisphere plant disjunctions: a window on tertiary land bridges and climate change? Ann. Bot. 98, 465–472. 10.1093/aob/mcl148 16845136 PMC2803576

[B45] Nosil P. (2012). Ecological Speciation (New York: Oxford University Press).

[B46] Orr M. R. Smith T. B. (1998). Ecology and speciation. Trends Ecol. Evol. 13, 502–506. 10.1016/S0169-5347(98)01511-0 21238408

[B47] Palme A. E. Su Q. Palsson S. Lascoux M. (2004). Extensive sharing of chloroplast haplotypes among European birches indicates hybridization among *Betula pendula*, *B. pubescens* and *B. nana* . Mol. Ecol. 13, 167–178. 10.1046/j.1365-294X.2003.02034.x 14653797

[B48] Papadopulos A. S. T. Baker W. J. Crayn D. Butlin R. K. Kynast R. G. Hutton I. (2011). Speciation with gene flow on Lord Howe Island. Proc. Nat. Acad. Sci. U. S. A. 108, 13188–13193. 10.1073/pnas.1106085108 PMC3156170 21730151

[B49] Petit R. J. Duminil J. Fineschi S. Hampe A. Salvini D. Vendramin G. G. (2005). Invited review: comparative organization of chloroplast, mitochondrial and nuclear diversity in plant populations. Mol. Ecol. 14, 689–701. 10.1111/j.1365-294X.2004.02410.x 15723661

[B50] Phillips S. J. Anderson R. P. Schapire R. E. (2006). Maximum entropy modeling of species geographic distributions. Ecol. Model. 190, 231–259. 10.1016/j.ecolmodel

[B51] Pickrell J. K. Pritchard J. K. (2012). Inference of population splits and mixtures from genome-wide allele frequency data. PLoS Genet. 8, e1002967. 10.1371/journal.pgen.1002967 23166502 PMC3499260

[B52] Polezhaeva M. A. Pimenova E. A. Tikhonova N. A. Korchagina O. S. (2018). Plastid DNA diversity and genetic divergence within *Rhododendron dauricum* s.l. (*R. dauricum* s.s., *R. ledebourii*, *R. sichotense* and *R. mucronulatum*; Ericaceae). Plant Sys. Evol. 304, 763–774. 10.1007/s00606-018-1508-1

[B53] Price A. L. Patterson N. J. Plenge R. M. Weinblatt M. E. Shadick N. A. Reich D. (2006). Principal components analysis corrects for stratification in genome-wide association studies. Nat. Genet. 38, 904–909. 10.1038/ng1847 16862161

[B54] Purcell S. Neale B. Todd-Brown K. Thomas L. Ferreira M. A. R. Bender D. (2007). PLINK: a tool set for whole-genome association and population-based linkage analyses. Am. J. Hum. Genet. 81, 559–575. 10.1086/519795 17701901 PMC1950838

[B55] Qiu Y. X. Qi X. S. Tao X. Y. Fu C. X. Naiki A. Comes H. P. (2009). Population genetic structure, phylogeography, and demographic history of *Platycrater argute* (Hydrangeaceae) endemic to East China and South Japan, inferred from chloroplast DNA sequence variation. Taxon 58, 1226–1241. 10.1016/j.jallcom.2006.08.275

[B56] Ribera I. Castro A. Diaz J. A. Garrido J. Izquierdo A. Jaech M. A. (2011). The geography of speciation in narrow-range endemics of the ‘Haenydra’ lineage (Coleoptera, Hydraenidae, Hydraena). J. Biogeogr. 38, 502–516. 10.1111/j.1365-2699.2010.02417.x

[B57] Rundell R. J. Price T. D. (2009). Adaptive radiation, nonadaptive radiation, ecological speciation and nonecological speciation. Trends Ecol. Evol. 24, 394–399. 10.1016/j.tree.2009.02.007 19409647

[B58] Rundle H. D. Nosil P. (2005). Ecological speciation. Ecol. Lett. 8, 336–352. 10.1111/j.1461-0248.2004.00715.x

[B59] Runemark A. Hey J. Hansson B. Svensson E. II (2012). Vicariance divergence and gene flow among islet populations of an endemic lizard. Mol. Ecol. 21, 117–129. 10.1111/j.1365-294X.2011.05377.x 22129244 PMC5617136

[B60] Schluter D. (2000). The Ecology of Adaptive Radiation (Oxford: Oxford University Press).

[B61] Schluter D. (2001). Ecology and the origin of species. Trends Ecol. Evol. 16, 372–380. 10.1016/S0169-5347(01)02198-X 11403870

[B62] Seehausen O. (2006). Sympatric speciation: Why the controversy? Curr. Biol. 16, R333–R334. 10.1016/j.cub.2006.03.077 16682343

[B63] Sharma A. Poudel R. C. Li A. Xu J. C. Guan K. Y. (2014). Genetic diversity of *Rhododendron delavayi* var. *delavayi* (C. B. Clarke) Ridley inferred from nuclear and chloroplast DNA: implications for the conservation of fragmented populations. Plant Syst. Evol. 300, 1853–1866. 10.1007/s00606-014-1012-1

[B64] Shrestha N. Wang Z. H. Su X. Y. Xu X. T. Lyu L. Liu Y. P. (2018). Global patterns of *Rhododendron* diversity: The role of evolutionary time and diversification rates. Global Ecol. Biogeogr. 27, 913–924. 10.1111/geb.12750

[B65] Stamatakis A. Hoover P. Rougemont J. (2008). A rapid bootstrap algorithm for the RAxML web servers. Syst. Biol. 57, 758–771. 10.1080/10635150802429642 18853362

[B66] Sun Y. Surget-Groba Y. Gao S. X. (2016). Divergence maintained by climatic selection despite recurrent gene flow: a case study of *Castanopsis carlesii* (Fagaceae). Mol. Ecol. 25, 4580–4592. 10.1111/mec.13764 27447352

[B67] Tajima F. (1989). Statistical method for testing the neutral mutation hypothesis by DNA polymorphism. Genetics 123, 585–595. 2513255 10.1093/genetics/123.3.585 PMC1203831

[B68] Taylor E. B. Boughman J. W. Groenenboom M. Sniatynski M. Schluter D. Gow J. L. (2005). Speciation in reverse: Morphological and genetic evidence of the collapse of a three-spined stickleback (*Gasterosteus aculeatus*) species pair. Mol. Ecol. 15, 343–355. 10.1111/j.1365-294X.2005.02794.x 16448405

[B69] Tikhonova N. A. Polezhaeva M. A. Pimenova E. A. (2012). AFLP analysis of the genetic diversity of closely related *Rhododendron* species of the section *Rhodorastra* (Ericaceae) from Siberia and the Far East of Russia. Russ J. Genet. 48, 1153–1161. 10.1134/S1022795412100110 23270263

[B70] Vonlanthen P. Bittner D. Hudson A. G. Young K. A. M€uller, R. Lundsgaard -Hansen B. (2012). Eutrophication causes speciation reversal in whitefish adaptive radiations. Nature 482, 357–362. 10.1038/nature10824 22337055

[B71] Voroshilov V. N. (1982). Opredelitel rastenii sovetskogo DalhegoVostoka (Moscow: Nauka Press).

[B72] Wan T. F. Cao R. P. (1992). Middle Eocene-Early Pleistocene tectonic events and stress fields in China. GEOCIECE 6, 3.

[B73] Wang X. Li Y. Liang Q. Zhang L. Wang Q. Hu H. (2015). Contrasting responses to Pleistocene climate changes: a case study of two sister species *Allium cyathophorum* and *A. spicata* (Amaryllidaceae) distributed in the eastern and western Qinghai-Tibet Plateau. Ecol. Evol. 5, 1513–1524. 10.1002/ece3.1449 25897390 PMC4395180

[B74] Watanabe Y. Ichiro T. Shota S. Song J. S. Yamamoto S. Tomaru N. (2016). Population demographic history of a temperate shrub, *Rhododendron weyrichii* (Ericaceae), on continental islands of Japan and South Korea. Ecol. Evol. 6, 8800–8810. 10.1002/ece3.2576 28035270 PMC5192946

[B75] Webb W. C. Marzluff J. M. Omland K. E. (2011). Random interbreeding between cryptic lineages of the Common Raven: Evidence for speciation in reverse. Mol. Ecol. 20, 2390–2402. 10.1111/j.1365-294X.2011.05095.x 21518060

[B76] Wright S. II Gaut B. S. (2005). Molecular population genetics and the search for adaptive evolution in plants. Mol. Biol. Evol. 22, 506–519. 10.1093/molbev/msi035 15525701

[B77] Wright S. (1984). Evolution and the genetics of populations (Chicago: University of Chicago press).

[B78] Wu C. Y. Lu A. M. Tang Y. C. Li D. Z. (2003). The Families and Genera of Angiosperms in China (A Comprehensive Analysis) (Beijing: Science Press).

[B79] Yang Z. (2007). PAML 4: phylogenetic analysis by maximum likelihood. Mol. Biol. Evol. 24, 1586–1591. 10.1093/molbev/msm088 17483113

[B80] Zachos J. Pagani M. Sloan L. Thomas E. Billups K. (2001). Trends, rhythms, and aberrations in global climate 65 Ma to present. Science 292, 686–693. 10.1126/science.1059412 11326091

[B81] Zhang L. Xu P. Cai Y. Ma L. Zhang C. Gao Q. (2017). The draft genome assembly of *Rhododendron delavayi* Franch. var. *delavayi* . Gigascience 6, 1–11. 10.1093/gigascience/gix076 PMC5632301 29020749

[B82] Zou X. H. Yang Z. Doyle J. J. Ge S. (2013). Multilocus estimation of divergence times and ancestral effective population sizes of *Oryza* species and implications for the rapid diversification of the genus. New Phytol. 198, 1155–1164. 10.1111/nph.12230 23574344

