# Supplementary material for: A Genomewide Scan for Genetic Structure and Demographic History of Two Closely Related Species, Rhododendron dauricum and R. mucronulatum (Rhododendron, Ericaceae)
Source: Front Plant Sci. 2020 Jul 17;11:1093. doi: 10.3389/fpls.2020.01093 (PMC7380098; doi:10.3389/fpls.2020.01093)
Supplement: Supplementary file 1 [file DataSheet_1.pdf]

Supplementary Material

1 Supplementary Figures

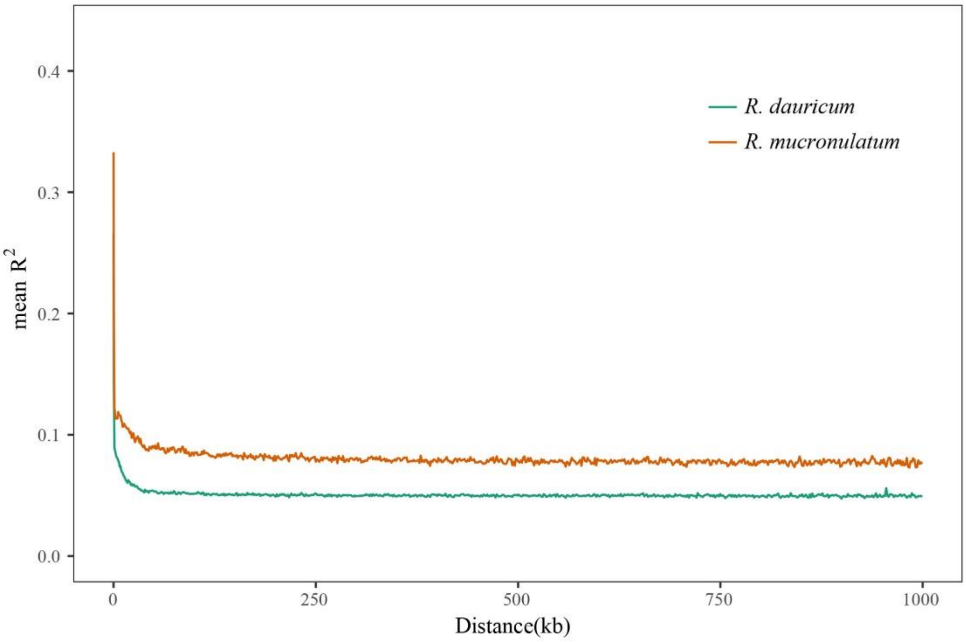

Figure S1. The LD-decay of *R. dauricum* and *R. mucronulatum* detected by PLINK in linkage disequilibrium (LD) analysis.

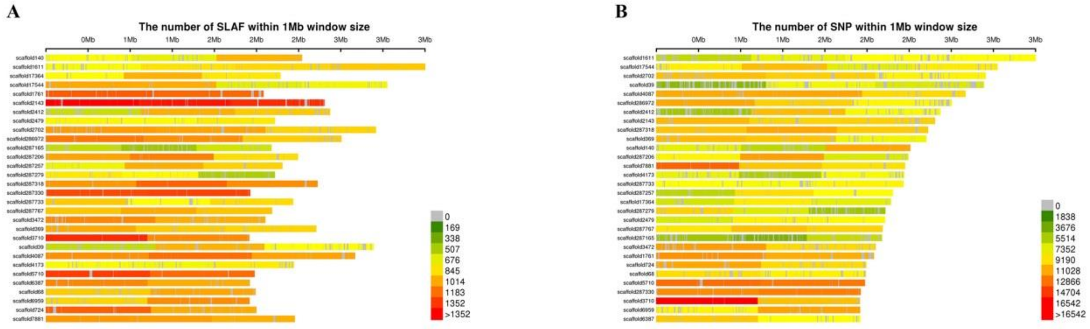

Figure S2. (A) Distribution diagrams of all SLAFs for *R. dauricum* and *R. mucronulatum* on the *R. delavayi* genome. (B) Distribution diagrams of all SNPs for *R. dauricum* and *R. mucronulatum* on the *R. delavayi* genome. According to the size of 1Mb, the genome was divided. The more the number of SLAF tags or SNPs in each window, the darker the color, the darker the area in the figure is the area where the SLAF tags or SNPs are concentrated.

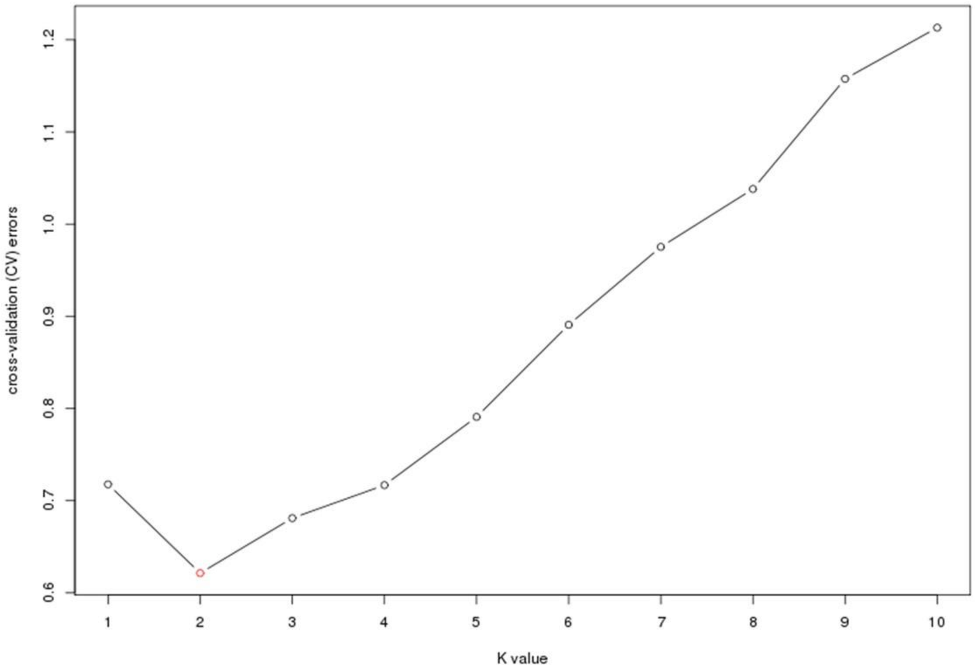

Figure S3. Cross-validation error rate for each K (1-10) value detected by Admixture for STRUCTURE analysis, k=2 was the optimal value.
